# Contribution-Based Resource Allocation for Effective Federated Learning in UAV-Assisted Edge Networks

**DOI:** 10.3390/s24206711

**Published:** 2024-10-18

**Authors:** Gang Xiong, Jincheng Guo

**Affiliations:** 1 The 30th Research Institute of China Electronics Technology Group Corporation, Chengdu 610000, China; 2 China Communications Construction Company, Ltd., Institute of Communication Technology, Dalian University of Technology, Beijing 100088, China; guojincheng@pdiwt.com.cn

**Keywords:** unmanned aerial vehicle, federated learning, resource allocation

## Abstract

This paper considers UAVs as edge computing nodes and investigates a novel network resource allocation method for federated learning within a three-layer wireless network architecture containing cloud, edges (UAVs), and clients. To address the issue of fair bandwidth resource allocation among clients participating in federated learning, a contribution calculation strategy based on the Shapley value (SV) used as the weight for model aggregation is proposed. On this basis, a client selection and wireless resource allocation method based on model contribution is further designed. By reducing the training and aggregation frequency of the low-contribution clients during the asynchronous aggregation phase, the limited bandwidth resources are allocated to high-contribution clients, thus improving the convergence speed and accuracy of the global model. Simulation experiments demonstrate that the proposed method can significantly reduce the system delay and total energy consumption with gains between 15% and 50% while also improving the final accuracy of the global model by 0.3% and 2% on both short-term and long-term perspectives, respectively.

## 1. Introduction

The next-generation 6G wireless networks need to adapt to unprecedented dynamic heterogeneous environments, promptly respond to network connection requests from resource-constrained heterogeneous terminal devices such as unmanned aerial vehicles (UAVs), and provide high-quality intelligent services such as sensing and computing [1]. In recent years, UAVs have been widely used in scenarios such as remote healthcare, environmental monitoring, emergency communications, and target identification due to their flexibility and mobility [2]. They can effectively collect data and provide communication and computational services in edge networks [3]. UAV-assisted edge networks based on federated learning can adapt to various network environments and data distribution scenarios, addressing practical issues in applications. For example, in remote healthcare consultations, they can prevent privacy leaks caused by centralized data processing; in environmental monitoring, they can efficiently collect sensor data from remote areas and process them in real time; in image-based target recognition, they can reduce the communication costs associated with data transmission [4,5]. The prerequisite for realizing these applications is a high-performance UAV-assisted federated learning architecture and the design of reasonable communication resource allocation algorithms [6,7].

In the two-layer federated learning system with a cloud–clients architecture [8], as the number of clients increases, the uplink load also increases, leading to network congestion and reduced uplink communication capacity per client. This decline in communication efficiency further affects the overall training performance of machine learning models. In contrast, in the two-layer federated learning system with edges–clients architecture, the number of clients that can participate in federated learning training is limited by the coverage area of the edge base station. The low number of user samples similarly has a negative impact on the overall performance [9,10]. To alleviate the significant communication pressure on cloud server while ensuring that the system can utilize more data, a three-layer federated learning architecture can be established by introducing a UAV-assisted edge layer between the cloud and clients [11]. By leveraging the intermediary characteristics of the edge layer, local models can be pre-aggregated at the edge, reducing the data volume for model updates by an order of magnitude. However, this architecture still faces multiple challenges, such as heterogeneity and fairness [12]. Designing a three-layer federated learning system that can meet the needs of heterogeneous devices, operate efficiently within limited spectrum resources, achieve rapid convergence, and enhance final performance is a critical focus of current research.

In traditional federated learning schemes, clients usually receive the same global model regardless of their individual contributions, neglecting the fairness of collaboration. Lyu et al. [13] designed a reputation mechanism where clients with low reputation values are prohibited from participating in aggregation, allowing participants to converge to different models to achieve fairness. Only by reasonably balancing each client’s contribution can a good global model be trained while incentivizing participants to provide high-quality data for training. Sim et al. [14] proposed a collaborative machine learning method that uses a reward evaluation approach based on the Shapley value (SV) and the information gain of model parameters to train multi-source data and build high-quality models. Song et al. [15] introduced an effectiveness measure based on the SV as a contribution index, using the intermediate results of federated learning to approximate model reconstruction on different combinations, thereby avoiding additional training. In addition to incentivizing high-quality data nodes to participate in collaborative training, the selection of underperforming clients can also be optimized. Sultana et al. [16] developed strategies to select certain clients for aggregation and adaptively adjust the frequency of local and global model updates to address efficiency and fairness issues in federated learning under resource constraints. Nguyen et al. [17] estimated client contributions based on the correlation between local and global updates, using the intelligent sampling of clients in each round of model training to optimize convergence speed.

Considering that federated learning is essentially a distributed machine learning architecture, its implementation in UAV-assisted wireless networks introduces new challenges [18]. To achieve faster and better aggregation of the global model, clients need to engage in multiple rounds of model interaction with the UAV during the training process, consuming significant amounts of time, energy, and bandwidth resources. Furthermore, bandwidth resources in a wireless communication environment are limited and valuable. The bandwidth shared under the same UAV constrains the communication time of its paired clients. The more clients participating in aggregation, the less bandwidth each client receives, reducing the number of aggregations possible within the same time frame and affecting the final convergence outcome [19]. During the training process, some devices may contribute more to the global model than others in a given round. If the contributions of each participant to the global model can be reasonably evaluated, they will be incentivized to provide higher quality data, and the overall training efficiency of federated learning can be improved. Therefore, it is imperative to design a contribution evaluation mechanism that ensures the final performance of the global model while minimizing the bandwidth waste and energy consumption of each client.

To address the challenges of fair selection and optimize the allocation of wireless resources, this paper focuses on the impact of client contributions on the convergence of the global model within a cloud–UAVs–clients three-layer federated learning architecture. The main contributions are as follows:

1. A contribution calculation strategy based on the SV is proposed and used as the weight during model aggregation. This method addresses the challenge to estimate the contribution of each client; thus, a client contribution analysis method is proposed by exploring the similarity among client models, edge (UAV) models, and the global model to rank clients’ potential contributions.
2. A wireless resource allocation algorithm based on client contribution is designed to reduce the aggregation opportunities for some low-contribution clients during the asynchronous aggregation phase. High-contribution clients will thus receive more training and aggregation opportunities. From a short-term perspective, this algorithm significantly reduces system delay and the total energy consumption of clients while achieving the given target accuracy. From a long-term perspective, it improves the final accuracy of the global model and achieves the state of the art.

## 2. System Model

**Figure 1:**
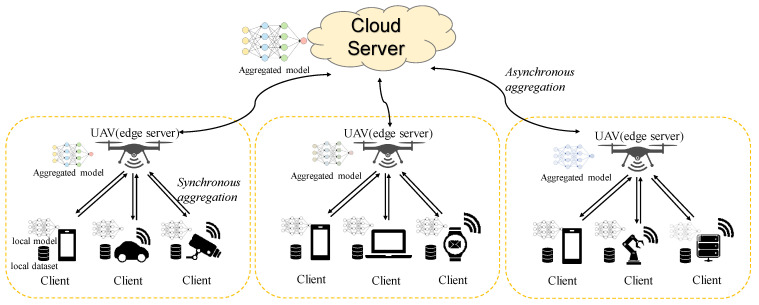
The three-layer federated learning architecture of clients–UAVs-cloud.

The three-layer federated learning architecture of clients–UAVs-cloud is shown in Figure 1. Let us assume that there are *K* clients in a three-layer network architecture, represented as $C=\left\{c_{1},c_{2},\ldots ,c_{K}\right\}$, where each client’s dataset is denoted by $D=\{D_{1},D_{2},\ldots ,D_{i},\ldots ,D_{K}\}$. There are *N* UAVs used for edge services, represented as $E=\left\{e_{1},e_{2},\ldots ,e_{N}\right\}$, and one cloud server [20].

If client $c_{i}$ is paired with UAV $e_{j}$, let $a_{i,j}=1$; otherwise, let $a_{i,j}=0$. Then, the set of clients paired with UAV $e_{j}$ can be denoted by $K_{j}=\left\{i\in N|a_{i,j}=1\right\}$. In this framework, the model aggregated by the UAV after aggregating the client models $W_{c}$ is referred to as the UAV edge model $W_{e}$, while the model aggregated by the cloud is referred to as the global model $W_{cloud}$. When the number of updates reaches the preset $\tau_{1}$ times, the clients send their models to their paired UAVs for aggregation. After $\tau_{2}$ executions, the UAVs send their edge models to the cloud for global aggregation. The entire process requires collaboration among the clients, UAVs, and cloud. In this paper, each communication with the cloud is considered one training round *T*, and a total of $T^{\prime }$ rounds are executed. The specific steps are as shown below.

1. Client Local Training: When the number of training rounds has not reached $\tau_{1}$, client $c_{i}$ trains the local model on its own data by using gradient descent. If $\tau_{1}$ is reached, the client transmits its updated model, which has undergone $\tau_{1}$ updates, to its paired UAV $e_{j}$.
2. UAV Edge Aggregation: UAV $e_{j}$ generates the edge model by aggregating the models of its paired clients by using the following aggregation method:
  (1) $$W_{e_{j}}^{T,t}=\frac{\sum_{i\in K_{j}}D_{i}\times W_{c_{i}}^{T,t}}{\sum_{i\in K_{j}}D_{i}}.$$
  If *t* has not reached the set rounds $\tau_{2}$, the UAV sends this edge model to its paired clients. If $\tau_{2}$ rounds are reached, the UAV transmits the aggregated edge model to the cloud.
3. Cloud Aggregation: Let the set of UAVs participating in the aggregation be Π. The cloud generates the global model by aggregating the edge models within the set by using the following method:
  (2) $$W_{cloud}^{T}=\frac{\sum_{j\in \Pi }\left(\sum_{i\in K_{j}}D_{i}\right)\times W_{e_{j}}^{T,t=\tau_{2}}}{\sum_{j\in \Pi }\sum_{i\in K_{j}}D_{i}}.$$
4. Model Update: The cloud sends the aggregated global model $W_{cloud}^{T}$ to the UAVs, which then transmit the global model to their paired clients. The clients use this global model as the initial model for the next round of training.

The UAV communication channel model at time *t* is represented as a combination of large-scale fading and small-scale fading, expressed as

(3) $$h_{t}^{ij}=\sqrt{\beta_{0}{\left(d_{t}^{ij}\right)}^{-\tilde{l}}}g_{t}^{ij},$$

where $\beta_{0}$ is the reference channel gain at a distance of 1 m, $d_{t}^{ij}$ represents the path loss, $\tilde{l}$ denotes the path loss exponent, and $g_{t}^{ij}$ is the small-scale fading coefficient. Due to the presence of line-of-sight (LoS) paths in UAV communication channels, small-scale fading can be modeled by using Rician fading, expressed as

(4) $$g_{t}^{ij}=\sqrt{\frac{{\tilde{K}}_{t}^{ij}}{{\tilde{K}}_{t}^{ij}+1}}g+\sqrt{\frac{1}{{\tilde{K}}_{t}^{ij}+1}}\tilde{g},$$

where |g|=1 represents the LoS channel component, $\tilde{g}$ is a circularly symmetric complex Gaussian random variable with zero mean and unit variance, and ${\tilde{K}}_{t}^{ij}$ is the Rician factor for the channel between $c_{i}$ and $e_{j}$.

## 3. Client Contribution Calculation Based on the SV for the Cloud

The advantage of the SV analysis method is that it allocates benefits based on each member’s contribution rate [21]. Applying this method to three-layer federated learning not only shows the principle of fairness but also effectively avoids the limitations of using a single evaluation standard. Due to the exponential increase in computational complexity of the SV analysis method with the number of clients, deploying this method in any environment will lead to significant time consumption issues. Therefore, this paper focuses on two main challenges: how to reasonably measure the contribution of each client and whether measuring the contribution will introduce additional computational overhead, thereby increasing the convergence time of the system model.

The method calculates client contributions as follows: When member *i* participates in a coalition *Q*, there are (|Q|−1)! possible permutations, where |Q| represents the number of members in coalition *Q*. The remaining (n−|Q|) members have (n−|Q|)! permutations. The weight of member *i* in the overall benefit distribution equals the number of different permutation combinations involving this member divided by the total number of permutations of *n* members, expressed as $\frac{(|Q|-1)!(n-|Q|)!}{n!}$. The marginal contribution that member *i* creates by participating in different coalitions can be expressed as v(Q)−v(Q∖{i}). Thus, the benefit that member *i* receives from the total benefit v(N) is

(5) $$sv_{i}=\sum_{Q\in N}\frac{(|Q|-1)!(n-|Q|)!}{n!}\times \left[v\left(Q\right)-v\left(Q\setminus \left\{i\right\}\right)\right],$$

where v(Q) represents the benefit obtained by the coalition *Q* and Q∖{i} denotes the *Q* with element *i* removed.

Let us assume that there are *K* clients and *N* UAV edge nodes (K>N). The calculation of the SV can be divided as follows: In the *T*-th round of global aggregation, the cloud receives edge models from *N* UAVs and calculates their SVs according to Formula (5), resulting in $SV_{edges}^{T}=\left\{sv_{e_{1}},sv_{e_{2}},\ldots ,sv_{e_{N}}\right\}$. Then, during the *t*-th asynchronous aggregation, the UAVs calculate the SVs of the models from its paired M=K/N clients, resulting in $SV_{clients}^{T,t}=\left\{sv_{c_{1}},sv_{c_{2}},\ldots ,sv_{c_{M}}\right\}$. The computational load on the cloud is 2N, while the additional computational load on the UAVs is 2K/N, which has a minimal impact on the overall federated learning delay.

As training progresses, the changes in the global model’s performance tend to decrease, leading to varying ranges of SVs in different rounds. Evaluating contribution based solely on the current round would result in the loss of information from previous training. Therefore, the concept of accumulated SV (ASV) is introduced for calculation:

(6) $$asv_{i}^{T}=\left\{\begin{array}{c} \rho *sv_{i}^{T}+\left(1-\rho \right)*sv_{i}^{T-1},T>0\\ sv_{i}^{T},T=0 \end{array}\right.,$$

where ρ is used to adjust the timeliness of the contribution. This approach captures the contributions of the UAVs from the beginning of training to the current round.

Beyond determining the contribution of each client model to the UAV model and the UAV model to the global model, it is essential to use this value to guide the convergence of federated learning, so we introduce a quality weight ω. UAVs with a negative Shapley contribution are considered to negatively impact the global model and are directly excluded from participating in the current round of aggregation, with their weight set to zero. For UAVs with a positive Shapley contribution, the values are normalized and used as the weight ω for aggregation in round *T*.

(7) $$\omega_{i}^{T}=\left\{\begin{array}{c} \frac{asv_{i}}{\sum_{i=0,a\;sv_{i}\geq 0}^{i=K}asv_{i}},a\;sv_{i}\geq 0,\\ 0,a\;sv_{i}<0. \end{array}\right..$$

The process from the cloud to the UAVs can be exemplified by Algorithm 1. The same applies to the process from the UAVs to its paired clients.

**Algorithm 1:** Aggregation strategy based on SV calculation.

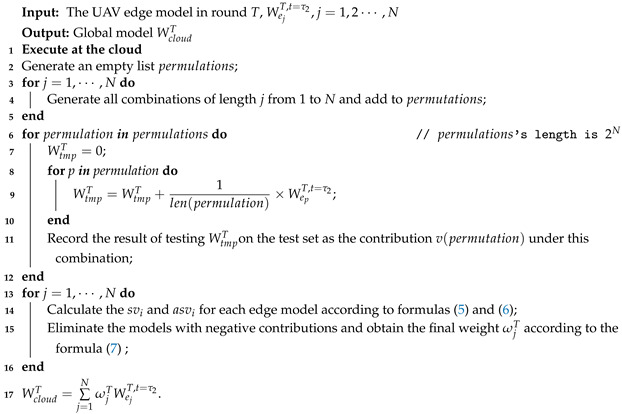



## 4. Client Contribution Calculation Based on Model Similarity for UAVs

Due to the high computational load, the SV method is generally used on the cloud. We further consider methods for calculating contributions for UAVs.

Traditional similarity determination methods usually involve the inner product or cosine similarity of vectors. If the inner product between the global model update gradient $\nabla f(w^{t})$ and the local training gradient of the client $\nabla F_{i}\left(w^{t}\right)$ is negative, i.e., if $\langle \nabla f\left(w^{t}\right),\nabla F_{i}\left(w^{t}\right)\rangle <0$, the device actually harms the performance of the global model. Cosine similarity is essentially a variation on the inner product, being the ratio of the inner product of vectors to the product of their magnitudes, thus capturing the similarity between vectors.

According to the semi-asynchronous framework, normal model aggregation is performed during the synchronous phase. After this phase, the UAV calculates the similarity of the client models it has received. While the UAV sends the models to the cloud, it simultaneously performs $t^{\prime }$ rounds of asynchronous aggregation with the clients. In the *T*-th round of global aggregation, let the local model trained by client $c_{i}$ during the *t*-th ($t<t^{\prime }$) asynchronous aggregation be denoted by $W_{c_{i}}^{T,t}$ and the edge sub-model obtained by the paired UAV during the synchronous aggregation be denoted by $W_{e_{j}}^{T,t=0}$. Let us assume that the data distribution of clients under each UAV’s coverage is Non-IID, while the data distribution among different UAVs can be approximated as IID [22].

Our goal is to improve the accuracy of the global model. Therefore, we need to obtain the update direction $d_{1}$ of the client model relative to the global model and the update direction $d_{2}$ of the client model relative to the UAV edge model for the current round and then calculate the cosine similarity between them:

(8) $$\mathrm{sim}\left(d_{1},d_{2}\right)=\frac{\langle d_{1},d_{2}\rangle }{\left|d_{1}\right|\cdot \left|d_{2}\right|}=\frac{\langle W_{c_{i}}^{T,t}-W_{cloud}^{T-1},W_{c_{i}}^{T,t}-W_{e_{j}}^{T,t=0}\rangle }{\left|W_{c_{i}}^{T,t}-W_{cloud}^{T-1}\right|\cdot \left|W_{c_{i}}^{T,t}-W_{e_{j}}^{T,t=0}\right|}.$$

After the cosine similarities $SIM_{e_{j}}=\left\{sim_{1},sim_{2},\ldots ,sim_{M}\right\}$ of all clients within the coverage area of the UAV are collected, they are sorted in descending order. The greater the similarity, the closer it is to the global model, indicating a higher contribution. The process is detailed in Algorithm 2.

**Algorithm 2:** Client contribution ranking based on cosine similarity.

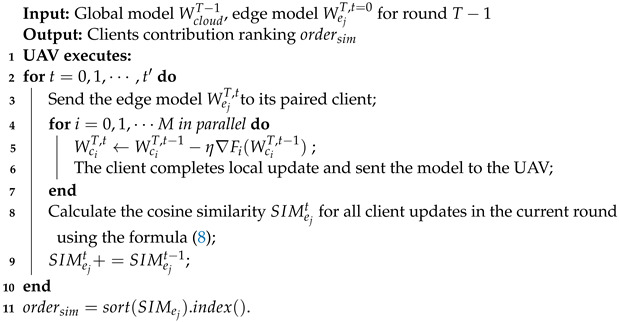



Intuitively, the difference between models represents the update direction. The idea of calculating the update direction $d_{1}$ of the client model $W_{c_{i}}^{T,t}$ relative to the global model $W_{cloud}^{T-1}$ comes from FedProx. Its purpose is to ensure that local updates do not deviate too much from the previous round’s global model, thereby reducing the impact of Non-IID while tolerating system heterogeneity and limiting local updates. Similarly, calculating the update direction $d_{2}$ of the client model $W_{c_{i}}^{T,t}$ relative to the edge model $W_{e_{j}}^{T,t=0}$ aims to keep client model updates close to the UAV edge model updates. By calculating the similarity between these two update directions, we can measure the contribution of each client to the global model under the constraint of the paired UAV. Additionally, this method significantly reduces the computation time compared with directly calculating the SVs for UAVs.

## 5. Wireless Resource Allocation Scheme

Limited wireless network resources require that a wireless federated learning system determines which clients can occupy the wireless channel to upload model updates and allocates bandwidth to each client in every round. This section focuses on the rational allocation of bandwidth resources in each round based on the previously discussed contribution calculations, effectively utilizing asynchronous aggregation.

The bandwidth resource allocation scheme in this section essentially serves as a client selection strategy. The main reason for this is the limitation imposed by the three-layer asynchronous federated learning framework. If the same number of clients in each round are allocated different bandwidths, without adjusting the transmission frequency of the clients, their communication delays will inevitably vary. Since the asynchronous communication delay between clients and UAVs is determined by the maximum delay, this would result in a value exceeding the average delay under equal bandwidth allocation, thereby increasing system delay. Therefore, the bandwidth allocation method in this section adopts uniform distribution. Based on this, the main focus of this section is to study how to optimize client selection during the asynchronous aggregation phase within the given time frame, i.e., adjust whether each client *i* should participate in asynchronous aggregation in the *r*-th round of global aggregation, denoted by $\alpha_{r,i}^{a}$.

From a short-term perspective, to quickly achieve the specified accuracy of the global model, the entire training cycle should be divided into two phases, i.e., early and late stages, focusing on accelerating convergence and improving performance, respectively. First, the contribution of edge models in the previous round is evaluated by the cloud server, and each is assigned a weight by using the SV. Then, the UAV with the lowest SV in the previous round is excluded from aggregation. During the acceleration phase, the UAV with the highest SV computes model similarity for its paired clients according to Algorithm 2, selecting the top-ranked ${Top}_{1}$ client to participate in asynchronous aggregation. This ranking is updated in real time during each asynchronous aggregation. In the performance improvement phase, the top $K^{\prime }$ UAVs with the lowest SV from the previous round are further excluded from aggregation. The number of clients within each UAV involved in aggregation is reduced, allowing the ${Top}_{2}$ clients with the highest similarity rankings paired with the high-SV UAVs to have more communication and computation opportunities, thereby improving overall performance. This algorithm primarily considers the limited computational capabilities of UAVs, as calculating the SVs of the paired clients would result in additional computational delay. The details are outlined in Algorithm 3.

**Algorithm 3:** Wireless resource allocation based on client contribution.

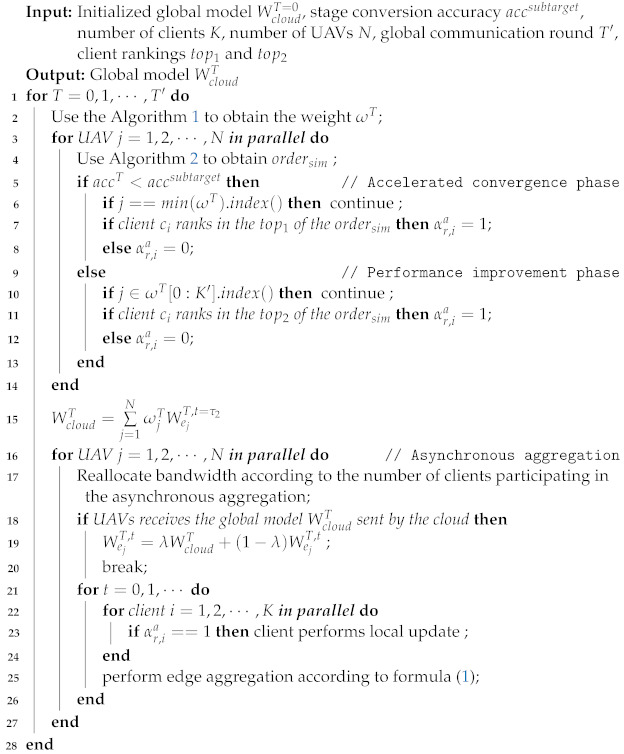



## 6. Simulations

The simulation experiment adopts a three-layer federated learning architecture consisting of 20 clients, 4 UAVs, and 1 cloud. The target area is divided into a 200 × 200 m grid, with each UAV hovering at a height of 15 m. Each UAV covers five ground clients, which are uniformly distributed within the grid. Each UAV and its paired clients are considered to be a cluster. The cloud is assumed to be far away from both the clients and the UAVs, and the time it takes for clients or UAVs to send models to the cloud is assumed to be approximately 10 times the communication time between the clients and the UAV. The parameters for the UAVs and wireless network scenario are shown in Table 1.

MNIST [23], CIFAR10 [24], and FashionMNIST [25] are used to perform image recognition tasks on the three-layer federated learning architecture we designed. On MNIST, the LeNet network is used for training [26]; on CIFAR10, a CNN network is used as the base encoder, consisting of three 3 × 3 convolutional layers, one 2 × 2 max-pooling layer, and one fully connected layer with a ReLU activation function. On FashionMNIST, a CNN is used as the base encoder, consisting of two 3 × 3 convolutional layers, one 2 × 2 max-pooling layer, and one fully connected layer with a ReLU activation function. The training settings are shown in Table 2. The deep learning software versions are Pytorch 2.0 and Python 3.8.

The synchronous aggregation strategy is FedAVG [27]; for asynchronous aggregation, the local training rounds $\tau_{1}^{a}$ are set to 40, and the asynchronous aggregation rounds between clients and UAVs $\tau_{2}^{a}$ are set to 8. The experiments are divided into the following two categories: one category uses similarity pairing and an improved loss function under the framework of Figure 1; the other category involves extending FEDAVG [28] to a three-layer federated learning architecture.

### 6.1. The Impact of Using SVs as Weights on Global Accuracy

We designed six configurations for comparison, which are as follows:

1. Configuration 1: Proposed framework (equal weight distribution);
2. Configuration 2: Proposed framework (cloud-to-UAV weighting according to SVs);
3. Configuration 3: Proposed framework (cloud-to-UAV and UAV-to-client weighting according to SVs);
4. Configuration 4: FEDAVG [28] (equal weight distribution);
5. Configuration 5: FEDAVG (cloud-to-UAV weighting according to SVs);
6. Configuration 6: FEDAVG (cloud-to-UAV and UAV-to-client weighting according to SVs).

**Figure 2:**
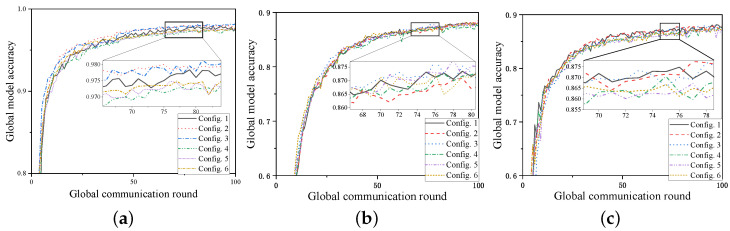
The curves of global model accuracy versus global training rounds under different configurations: (**a**) MINST. (**b**) CIFAR10. (**c**) FashionMNIST.

According to Figure 2, all six configurations converge with relatively good accuracy after 100 global communication rounds. However, as seen from the magnified box, the accuracy of our framework (configurations 1, 2, and 3) is significantly better than the FEDAVG framework (configurations 4, 5, and 6). Additionally, within the same framework, the accuracy achieved by using the SV method is noticeably better compared with the weight averaging method, i.e., configurations 2 and 3 are better than configuration 1, and configurations 5 and 6 are better than configuration 4. So, using the SVs of the UAVs and clients as weights for model aggregation in a three-layer federated learning model significantly improves global accuracy, whether in the classic FEDAVG framework or our framework. When SVs are used as weights for cloud-to-UAV or UAV-to-client (configurations 2, 3, 5, and 6) in the three datasets, the highest accuracy of the global model within 100 training rounds is 0.4% to 1% higher than that of the ordinary aggregation framework (configurations 1 and 4). This indicates that assigning higher weights to models with higher contributions, as measured by their local (or UAV) model contributions, can improve the accuracy of the global model and incentivize clients to provide more valuable data during federated learning training. Moreover, using SVs as weights can significantly enhance the convergence efficiency of the global model in the early stages. On the MNIST dataset, the number of global rounds required to reach 95% accuracy is reduced by five–seven rounds. Since the communication delay and energy consumption per round of training are constant, this results in approximately 20% savings in system delay and client energy consumption compared with the original framework. Similarly, there are about 3% to 10% reductions in delay and energy consumption on the CIFAR10 and FashionMNIST datasets. The specific data are shown in Table 3.

**Figure 3:**
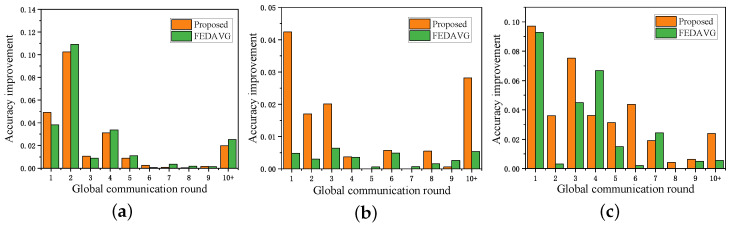
Based on (**a**) MINST, (**b**) CIFAR10, and (**c**) FashionMNIST, the accuracy improvement of the global model after using SVs as aggregation weights.

For each round of global communication, the increase in global model accuracy before and after using SVs as weights for aggregation is shown in Figure 3. In the first 10 rounds, applying SVs as the aggregation weights at the UAV results in a significant increase in global model accuracy, accounting for approximately 80% of the total improvement across all rounds. In contrast, the total improvement from the 10th to the 100th round (represented by the “10+” column) is relatively small. This leads to the conclusion that SVs are highly effective for accelerating aggregation in the early stages. In the later stages, the contribution to improvement diminishes as the local models converge.

### 6.2. Experimental Comparison of Various Resource Allocation Methods

This section compares the effects of various resource allocation methods on aggregation performance and energy consumption. To ensure fairness, the experiments are all based on configuration 2. Although the results of configuration 3 are superior, its longer computation time at the UAV increases delay. The comparison is made from two perspectives: short-term metrics, which include system delay and total client energy consumption when each strategy reaches different target accuracies, and long-term metrics, which include the highest accuracy of the global model. The comparison involves the following bandwidth allocation methods:

1. Method 1: Full Aggregation. All clients participate in aggregation;
2. Method 2: Random Aggregation. Clients participate in aggregation according to a random proportion frac;
3. Method 3: Round Priority. Clients that did not participate in the previous round are prioritized, with the remaining clients being chosen randomly;
4. Method 4: “Later is better” [29]. The client participation ratio is allocated from low to high across all training stages;
5. Method 5: FedPNS [30]. Clients with higher local losses are preferentially selected.

These methods all ensure that the time for asynchronous aggregation is less than the time for UAVs to transmit parameters to the cloud. The client participation ratio frac for Methods 2, 3, and 5 is the same, set to 0.75. For Method 4, the participation ratio increases by five clients every 25 rounds. Method 5 introduces additional communication time with the cloud if it compares the gradient information of all clients in the current round. Therefore, it uniformly compares with the local loss from the previous round.

**Figure 4:**
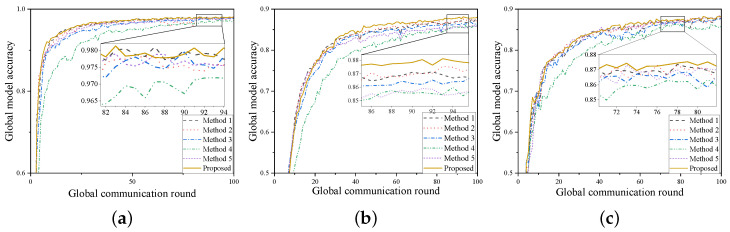
The curves of global model accuracy versus global training rounds under different resource allocation methods: (**a**) MINST. (**b**) CIFAR10. (**c**) FashionMNIST.

Figure 4 shows the variation in global model accuracy with global training rounds under different resource allocation strategies. It can be observed that the proposed algorithm is optimal in both convergence speed and final model accuracy. Method 1 does not select clients and allocates all resources equally to all clients. This approach is fair to all clients and can improve the final accuracy while reducing the total delay. However, since every client participates in training and asynchronous communication, it leads to excessively high energy consumption. Method 2 randomly selects clients, serving as a compromise strategy that balances global energy consumption and delay. It neither causes excessive energy consumption nor hampers convergence. Method 3 combines Method 1 and Method 2, incorporating some randomness while ensuring that each client has an equal chance to participate. However, the result shows that it does not inherit the advantages of either method. Method 4 assumes a fixed total number of clients selected throughout the federated learning process, with more clients participating in aggregation as training progresses. This results in significantly higher delay in short-term metrics compared with other methods. However, considering only long-term metrics and disregarding system delay, this method has higher feasibility. Method 5 performs poorly on the CIFAR10 dataset because, unlike the other two datasets, the data label distribution among different clients follows a Dirichlet distribution. This indicates that the method performs well only under specific data distributions. From a short-term perspective, to reach the specified accuracy, the proposed algorithm accelerates training and convergence by eliminating training opportunities for some low-contribution clients and reallocating bandwidth resources to other high-contribution clients. This approach leads to significant improvements in most short-term metrics, shortening the time by up to 60% and saving up to 40% in client energy consumption compared with other methods. From a long-term perspective, high-contribution clients inherently provide better training results. Under the same number of training rounds, our algorithm can increase the highest accuracy by an additional 0.3% to 2%. The specific values can be seen in Table 4.

### 6.3. Ablation Experiment

**Figure 5:**
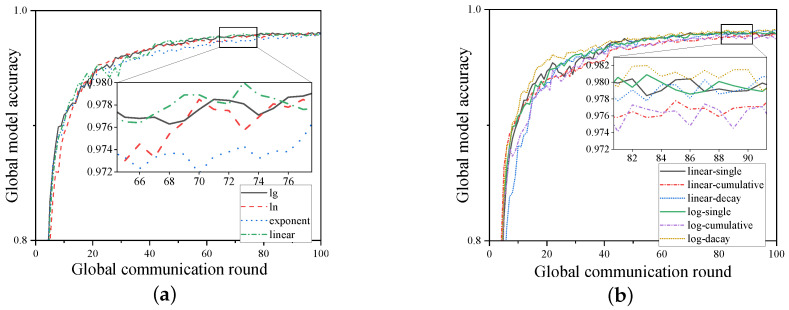
Analysis of the impact of different aggregation weight transformation functions on global accuracy on the MNIST dataset. (**a**) The impact of using linear, logarithmic, and exponential functions as weight transformation functions on the global model; (**b**) the impact of using logarithmic and linear functions as aggregation weight transformation functions on the global model under single-round, cumulative, and decay conditions.

In this section, ablation experiments are conducted on the scheme by using SVs as aggregation weights. Experiments are performed based on different forms of weight transformation functions, specifically linear, logarithmic, and exponential functions. This is because when the SVs of various UAVs differ significantly, the weight transformation directly determines whether this difference is further amplified or reduced. Experiments are conducted based on configuration 2 (the proposed framework + cloud-to-UAV weighting based on SVs), and Figure 5 shows the comparison of global model accuracy on the MNIST dataset, obtained through various weight transformation functions between the cloud and UAVs.

According to Figure 5a, using the exponential function as the weight transformation function yields the worst results, which is due to its steep increase. When there is already a difference in the SVs of the UAV models, the exponential function further enlarges the weight disparity, causing the weights to fail to accurately reflect the contribution proportions. As a result, the aggregated global model is biased toward the UAV model with the larger weight and fails to converge to the global optimal solution. In contrast, using linear and logarithmic functions as transformation functions correctly reflects the contribution proportions and even reduces the weight differences between different models to some extent, leading to the global model converging with better accuracy.

We further analyze the linear and logarithmic functions by conducting experiments in the following three scenarios: (1) single-round method, where only the SV from the previous round is used as the weight reference; (2) cumulative method, where the SVs from all previous rounds are accumulated and used as the weight reference; (3) decay method, where the SVs from all previous rounds are decayed by 10% during the training process and then used as the weight reference. The experimental results are shown in Figure 5b. It can be clearly observed that using the single-round method yields better performance on this dataset; however, the accuracy of the global model using the cumulative method is lower than that of other methods. This is because directly accumulating the SVs leads to an excessively large weight proportion for the UAVs that provided high-quality models in certain rounds, which interferes with the weight transformation of the SVs in other rounds. The decay method effectively mitigates this issue by reducing the influence of earlier rounds while still retaining the contribution information from those rounds, resulting in excellent performance as well.

## 7. Conclusions

Regarding the optimization of bandwidth resource allocation for three-layer federated learning when clients have homogeneous resources, this paper focuses on the impact of edge model contribution on the convergence of the global model. We propose an algorithm that selects and allocates resources to each client based on their contribution and model similarity. By eliminating the aggregation opportunities for some low-contribution clients during the asynchronous aggregation phase, high-contribution clients gain more training and aggregation opportunities. This algorithm reduces system delay and total client energy consumption for a given target accuracy. Experimental comparisons show that using the SVs of the participating UAVs or clients as weights for model aggregation significantly improves overall accuracy. Moreover, the client selection and bandwidth resource allocation algorithm performs well across three datasets, achieving system delay and energy consumption savings of 15% to 50% while also improving the final accuracy of the global model. The algorithm shows good performance from both short-term and long-term perspectives.

## Figures and Tables

**Table 1:** UAV and wireless network parameter settings.

CPU frequency of UAV $f_{i}$	1 × $10^{9}$ Hz
The cycles of training unit data $c_{i}$	20 cycles/bit
Capacitance coefficient $\frac{a_{i}}{2}$	2 × $10^{-28}$
Bandwidth *B*	1 × $10^{6}$ Hz
Client transmission power $p_{i}$	0.5 W
Channel gain at a distance of 1 m $\beta_{0}$	−60 dB
The power spectral density of Gaussian noise $N_{0}$	1 × $10^{-10}$ W
Path loss exponent $\tilde{l}$	2.4

**Table 2:** Training settings.

	MNIST	CIFAR10	FashionMNIST
Learning rate	0.01	0.1	0.01
Batch size	30	30	30
Hyperparameter γ	0.1	10	1
Hyperparameter μ	10	0.1	0.1
Total parameters	28,840	3,593,185	441,646

**Table 3:** The highest accuracy of the global model and the corresponding global rounds under different configurations.

		Config. 1	Config. 2	Config. 3	Config. 4	Config. 5	Config. 6
MNIST	Highest accuracy in 100 rounds	0.9778	0.9805	**0.9823**	0.9723	0.9748	0.9761
	Corresponding global rounds	26	**20**	**20**	27	24	22
CIFAR10	Highest accuracy in 100 rounds	0.8818	**0.8849**	0.8809	0.8767	0.8818	0.8815
	Corresponding global rounds	**45**	46	46	49	46	**45**
FashionMNIST	Highest accuracy in 100 rounds	0.8766	0.8805	**0.8812**	0.8721	0.8732	0.8754
	Corresponding global rounds	40	**36**	39	42	45	43

The numbers in bold indicate the maximum accuracy or the minimum global rounds in each row.

**Table 4:** The delay and total client energy consumption to reach the target accuracy under different resource allocation schemes. “-” indicates that the target accuracy was not reached. The highest accuracy refers to the maximum accuracy the global model can achieve after 100 rounds of global communication. The unit of time delay is seconds, and the unit of energy consumption is joules.

MNIST									
	Target Accuracy: 95%		Target Accuracy: 96%		Target Accuracy: 97%		Target Accuracy: 98%		Highest Accuracy
	Time	Energy	Time	Energy	Time	Energy	Time	Energy	
	Delay	Consumption	Delay	Consumption	Delay	Consumption	Delay	Consumption	
Method 1	459	2647	557	3342	753	4884	-	-	0.9787
Method 2	441	2106	647	3313	818	4361	-	-	0.9793
Method 3	521	2545	641	3311	846	4549	-	-	0.9778
Method 4	714	2534	896	3577	1197	5622	-	-	0.9729
Method 5	357	1607	513	2138	852	3968	-	-	0.9785
Proposed	**303**	**1577**	**384**	**1985**	**618**	**3219**	**1105**	**6379**	**0.9821**
**CIFAR10**									
	**Target Accuracy: 80%**		**Target Accuracy: 85%**		**Target Accuracy: 87%**		**Target Accuracy: 88%**		**Highest Accuracy**
	**Time**	**Energy**	**Time**	**Energy**	**Time**	**Energy**	**Time**	**Energy**	
	**Delay**	**Consumption**	**Delay**	**Consumption**	**Delay**	**Consumption**	**Delay**	**Consumption**	
Method 1	**42,146**	373,064	90,978	876,348	150,261	1,356,303	-	-	0.8757
Method 2	**42,146**	**252,494**	82,365	496,337	151,972	923,471	-	-	0.8739
Method 3	49,252	349,815	96,148	690,288	-	-	-	-	0.8675
Method 4	68,244	291,711	143,285	785,329	-	-	-	-	0.8602
Method 5	45,766	268,513	125,854	756,883	-	-	-	-	0.8602
Proposed	**42,146**	266,989	**72,014**	**424,287**	**117,131**	**662,492**	**153,755**	**855,467**	**0.8818**
**FashionMNIST**									
	**Target Accuracy: 80%**		**Target Accuracy: 85%**		**Target Accuracy: 87%**		**Target Accuracy: 88%**		**Highest Accuracy**
	**Time**	**Energy**	**Time**	**Energy**	**Time**	**Energy**	**Time**	**Energy**	
	**Delay**	**Consumption**	**Delay**	**Consumption**	**Delay**	**Consumption**	**Delay**	**Consumption**	
Method 1	6568	43,326	10,512	72,863	18,208	133,905	23,011	171,745	0.8816
Method 2	**5987**	**28,195**	11,182	54,857	17,323	88,089	-	-	0.8798
Method 3	6851	37,149	15,035	88,572	20,286	121,974	-	-	0.8763
Method 4	9117	31,239	16,433	72,682	-	-	-	-	0.8622
Method 5	6266	29,429	**10,479**	**50,718**	17,325	87,991	22,334	115,096	0.8833
Proposed	**5987**	28,467	10,955	54,260	**14,531**	**67,545**	**21,191**	**92,792**	**0.8844**

The numbers in bold indicate the minimum time delay or energy consumption, or the maximum accuracy in each column.

## Data Availability

The original contributions presented in the study are included in the article, further inquiries can be directed to the corresponding author.
